# Photosensitive Epilepsy and Polycystic Ovary Syndrome as Manifestations of MERRF

**DOI:** 10.1155/2020/8876272

**Published:** 2020-09-28

**Authors:** Josef Finsterer

**Affiliations:** Klinik Landstrasse, Vienna, Austria

## Abstract

**Objectives:**

Although endocrinologic involvement and epilepsy are frequent features of myoclonic epilepsy with ragged-red fibers (MERRF), polycystic ovary syndrome (PCOS) and photosensitive epilepsy have not been reported. *Case Report*. A 32-year-old female was diagnosed with MERRF at age 19 y upon presence of the four canonical features and the variant m.8344A > G in *MT-TK* (*tRNA (Lys)*) (blood heteroplasmy rate: 50%). She experienced recurrent photosensitive focal and generalised seizures since age 19 y, which could be triggered by flickering light or by looking at small stones, leaves, or dirty snow on the ground. Since the last 42 months, she was seizure-free upon levetiracetam (4000 mg/d), clonazepam (1.5 mg/d), and topiramate (25 mg/d). Additionally, she suffered from secondary amenorrhoea since adolescence. She was married between ages 19 y and 25 y but did not get pregnant. PCOS was diagnosed and treated with desogestrel plus estradiol. Nonetheless, the course was progressive, particularly with regard to ataxia, myocloni, and myopathy.

**Conclusions:**

The phenotypic spectrum of MERRF is broader than anticipated and may additionally include PCOS and photosensitive epilepsy. PCOS in MERRF may respond to hormone substitution and photosensitive epilepsy to levetiracetam, clonazepam, and topiramate.

## 1. Introduction

Myoclonic epilepsy with ragged-red fibers (MERRF) is a rare syndromic mitochondrial disorder (MID) due to currently 26 mutations in 15 different genes [1]. The most frequent of these mutations is the variant m.8344A > G in *MT-TK* (*tRNA (Lys)*) [1]. Although MERRF is clinically characterised by the four canonical features, ataxia, epilepsy, myoclonus, and myopathy [1–3], it is a multisystem disorder with a much broader phenotypic spectrum (MERRF-plus) [1, 2]. Organs affected in addition to the brain and the muscle in MERRF-plus include the peripheral nerves, eyes, ears, heart, gastrointestinal tract, or the endocrine organs [1]. Although involvement of the brain and endocrine system in MERRF-plus is well appreciated [3, 4], polycystic ovary syndrome (PCOS) and photosensitive epilepsy have not been reported as a phenotypic manifestation of MERRF-plus.

## 2. Case Report

The patient is a 32-year-old Caucasian female, of height 166 cm and weight 50 kg, with uneventful early development who became noteworthy at age 7 y because of poor school performance due to impaired memory and concentration. She had nocturnal enuresis until age 14 y and menarche at age 13 y with normal periods thereafter. At age 17 y, recurrent, spontaneous myocloni of all four limbs began. A first generalised tonic-clonic seizure occurred at age 19 y. The postictal period was characterised by severe muscle weakness and muscle aching during 24 h. Electroencephalography (EEG) recording revealed epileptiform discharges in the frontocentral regions under hyperventilation and a decreased photoparoxysmal threshold. Valproic acid (VPA) was begun.

**Figure 1:**
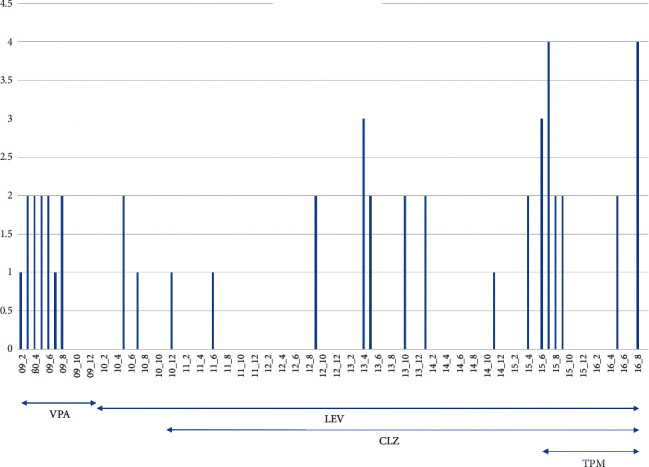
Frequency of focal and generalised seizures since onset at age 19 y in the index patient (1): focal seizure, (2): one generalised seizure, (3): two generalised seizures, (4): three generalised seizures per day).

**Figure 2:**
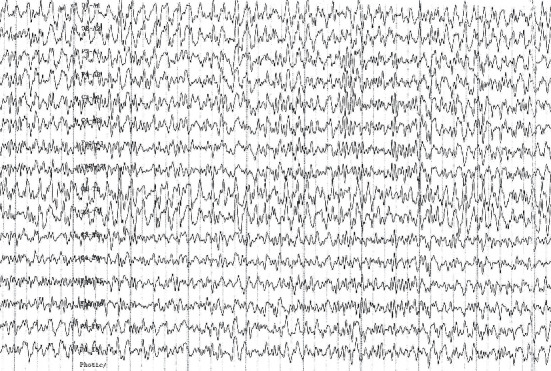
EEG under flickering light at age 24 y showing generalised, bilaterally synchronous spike-wave and polyspike-wave complexes.

Despite VPA, seizures recurred with a frequency of 1-2 seizures/month. In addition to generalised seizures, she rarely experienced focal seizures (Figure 1). Seizures could be triggered by flickering light or by looking at small stones, leaves, or dirty snow on the ground. Walking safely for 1 km with one stop was possible only after sunset. Nerve conduction studies revealed a mixed axonal/demyelinating neuropathy. Electromyography was myogenic. MRI of the brain at ages 19 y, 21 y, and 28 y revealed cerebellar atrophy exclusively. At age 20 y, VPA was replaced by levetiracetam (LEV) 3000 mg/d. Genetic work-up at age 21 y revealed the variant m.8344A > G with a heteroplasmy rate of 50% in blood lymphocytes. EEG at age 24 y revealed bilaterally synchronous spike-wave complexes in the occipital area. Flickering light triggered generalised, bilaterally synchronous spike-wave and polyspike-wave complexes (Figure 2). The patient had been married between ages 19 y and 25 y but never became pregnant. Work-up for infertility at age 20 y revealed a PCOS for which she received desogestrel (150 *μ*g/d) + estradiol (20 *μ*g/d) twice a year for three months since age 26 y. Since age 27 y, ataxic gait worsened such that she required support from another person for walking. Elevated serum lactate levels up to double the upper reference limits were noted for the first time. At age 27 y, topiramate (TPM) was added. Seizures had not recurred during 42 months prior to the last follow-up at age 32 y (Figure 1).

**Figure 3:**
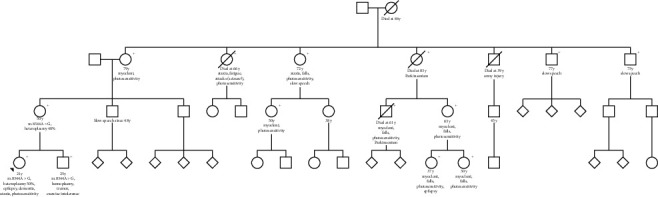
Pedigree of the index patient's family (the “+” indicates that the individual was clinically affected).

The family history was positive for easy fatigability (mother (48 yo), brother (26 yo), great aunt from mother's side), photosensitivity (mother, grandmother from mother's side, 2 great aunts from mother's side, 2 aunts and 1 uncle from mother's side), hand tremor (brother), myocloni (grandmother from mother's side, 2 aunts, 1 uncle, 2 cousins from the mother's side), seizures (1 great aunt from the mother's side, 1 cousin), Parkinsonism (1 great aunt from the mother's side, 1 uncle), ataxia (2 great aunts from the mother's side), and hypothyroidism (mother) (Figure 3). The mother felt unpleasant with flickering light and when looking at many stones, leaves, or dirty snow on the ground. Both mother and brother of the index patient carried the m.8344A > G variant, the mother had a heteroplasmy rate of 40% and the brother was homoplasmic. Clinical neurologic exam of the index patient at age 32 y revealed cognitive impairment, ataxia, photosensitivity, dysarthria, uncoordinated speech, myocloni of upper limbs, generalised wasting, absent tendon reflexes, and gait disturbance. The current medication included LEV (4000 mg/d), clonazepam (CLZ) (1.5 mg/d), TPM (25 mg/d), coenzyme-Q (200 mg/d), L-carnitine (2 g/d), and vitamin-E (400 mg/d).

## 3. Discussion

The presented patient is interesting for the following aspects. First, the patient had MERRF, which is still a rare MID syndrome. MERRF was diagnosed upon presence of all four canonical features and the m.8344A > G variant. The index patient had inherited the disease from her mother who was only mildly affected. The low heteroplasmy rate of 50% does not exclude pathogenicity, as it had been determined only in blood.

Second, the patient had photosensitive epilepsy, which has not been reported in MERRF. It is characterised by the occurrence of seizures after exposure to certain visual stimuli. Many patients experience an aura prior to onset of the seizure or feel odd sensations before the seizure occurs [5]. Interestingly, the same triggers that induced epilepsy in the index patient induced only an unpleasant feeling in the mother. Although the mother had never recorded an EEG, it is conceivable that the unpleasant feeling actually represents focal seizures.

Third, the index patient had PCOS, which has not been reported in MERRF. PCOS was speculated to have been triggered by VPA, as has been previously reported [6], but the question if VPA truly causes PCOS is still under debate [7, 8]. A strong argument against VPA as the trigger of PCOS is that PCOS has been reported in MIDs other than MERRF and not under VPA [9, 10]. Thus, it is more likely that PCOS in the index patient was a manifestation of the underlying MID than a side effect of VPA. A further argument against VPA is that many MID patients received VPA without developing PCOS. Whether VPA enhanced the preexisting endocrine problem remains speculative.

Fourth, homoplasmy of the m.8344A > G variant has been only rarely reported [11, 12]. Whether patients carrying a homoplasmic m.8344A > G variant are more severely affected than heteroplasmic patients is unknown but the few cases available suggest that onset is earlier and the phenotype is more severe in homoplasmic than heteroplasmic patients.

Limitations of the study are that no muscle biopsy had been taken, that heteroplasmy was determined only in blood lymphocytes, that no prospective investigations for multisystem involvement had been carried out, that first-degree relatives were not systematically investigated, and that the progression of the disease had been only faultily monitored.

Overall, this case shows that the phenotypic spectrum of MERRF-plus is broader than anticipated and may additionally include PCOS and photosensitive epilepsy. PCOS in MERRF may respond to hormone substitution and photosensitive epilepsy to LEV, CLZ, and TPM.
